# Much have I travel’d in the realms of gold^1^

**DOI:** 10.3201/eid1710.AC1710

**Published:** 2011-10

**Authors:** Polyxeni Potter

**Affiliations:** Centers for Disease Control and Prevention, Atlanta, Georgia, USA

**Keywords:** art science connection, emerging infectious diseases, art and medicine, Rembrandt van Rijn, Aristotle with a Bust of Homer, fungus, fungal infections, portraiture

**Figure:**
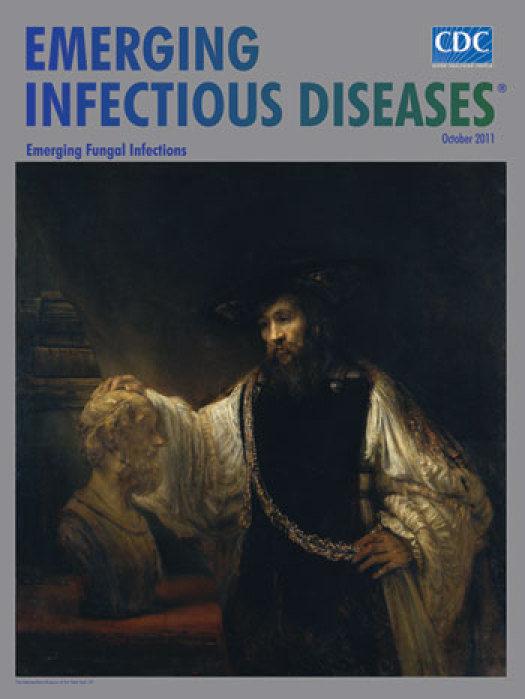
**Rembrandt van Rijn (1606–1669) *Aristotle with a Bust of Homer* (1653) Oil on canvas (143.5 cm × 136.5 cm).** The Metropolitan Museum of Art, New York, NY

“Know thyself” resonated with Rembrandt van Rijn. More than any other artist of his caliber at any time, he explored his own image in as many as 90 self-portraits, some 60 of them paintings, an extraordinary record of self-examination. He was so frank with his depictions he could not have been motivated by narcissism. He may have taken up portraiture for its connection to history painting, a lifelong interest. Portraits were very popular in the commercial market of his day, and his were much sought after in Holland. Whatever the motivation, his self-portraits captured much more than physical features. From youth to ripe old age they amounted to a spiritual autobiography, and since he never strayed more than a few miles from his native Leiden, the journey of discovery was an inward one.

Rembrandt’s life has been shrouded in mystery, largely because no written records exist beyond the usual certificates of birth, baptism, marriage, and death. He left no journal, and seven surviving letters from his hand concern routine transactions. But for an inventory of his possessions when near the end of his life he declared himself insolvent, the great artist left few clues about himself, save in his art, a brilliant legacy of more than 2,300 works, among them the famed *Anatomy Lesson of Dr. Tulp*, *Night Watch*, some of his self-portraits, and *Aristotle with a Bust of Homer*, on this month’s cover.

*Aristotle with a Bust of Homer* was commissioned by Don Antonio Ruffo, Sicilian nobleman, art collector, and patron of Rembrandt, without specific guidance, except to paint a philosopher. The meeting of the minds ensemble that ensued was the painter’s idea. A mixture of history and myth, the composition contains Rembrandt hallmarks: simplicity, quiet, character, empathy. While two figures are clearly present, a third one, Alexander the Great, appears indirectly, on the ornate pendant worn by Aristotle.

The philosopher is portrayed in his study as a distinguished figure, clad in finery reminiscent of the Renaissance. A certain social rank, the markings of which appear in other works by Rembrandt, including some self-portraits, is present in the elegant attire and the sensitive ringed hands. Though not a military man, Aristotle seems decorated, the gold chain and medallion bestowed upon him by the warrior prince displayed prominently. Secure in his own stature, Aristotle seems lost in thought. He rests one hand on the bust as he casts brooding eyes on antiquity’s celebrated poet, “deep-brow’d” Homer, a figure much admired by Aristotle and revered by Alexander, Aristotle’s pupil, who carried everywhere he went a copy of The Iliad, annotated by his tutor.

This imaginary meeting of three ancient historical figures, a gathering of genius, shows not only the artist’s inventiveness and technical brilliance but also his thoughts on the subject. Asked to paint a generic philosopher, he was intrigued by his own choice, and “Like some watcher of the skies/when a new planet swims into his ken,” he did much more.

Homer is a legend. The exact period of his life has been debated, his very existence questioned. Skeptics have been so doubtful about him, it has been said in jest that the epic works were not created by him but by someone else with the same name. Still, Homer persists as poet of The Iliad and The Odyssey. Thought to have lived close to 3,000 years ago, he predated realistic portraiture. His image was invented much later and frequently copied, always sightless and bearded, often wearing a headband. Rembrandt likely relied on Hellenistic busts in his own collection for guidance.

The dark and stillness of the room and faint outline of books in back amplify the lighted face and figure of the philosopher. His depiction as a Renaissance man, be it artistic license or intentional anachronism, could not have been more apt. Aristotle knew and understood all that was known in his day, to which he contributed in spades. A man for whom no discipline was uninteresting or unattainable, he was as comfortable with the arts as he was with the sciences.

This extraordinary empirical man paused with humility in front of the revered poet, who explored the mysteries of the human heart. Homer’s world, a place of conflict and adversity against which humans were expected to show strength, courage, and perseverance paved the way for philosophy. His was too a world full of wonder and discovery: close calls, shipwrecks, natural catastrophes, lotus-eaters, Cyclops, Sirens, the bravest men, the most beautiful woman, the most faithful wife.

In Rembrandt’s portrait, the haunting eyes that surveyed the totality of human knowledge are unfathomable. Contemplation, the philosopher wrote in the Nicomachean Ethics, is the highest form of happiness, and of all pleasures in life, it is the most enduring and self-sufficient. Since the intellect (*νους*) is our most exalted attribute and what it grasps is the highest knowledge, contemplation must be the ultimate form of human activity. Equal to philosophical wisdom, it involves scientific understanding―the intuitive grasp of eternal first principles combined with demonstration.

Contemplation as guide to life has been interpreted in many ways. In the thousands of years since Homer, Aristotle, and Alexander, many have taken the philosopher’s call, and some have written modern Odysseys. More than 70,000 species of fungi alone have been described since Aristotle classified living things into animals and plants. His theory of spontaneous generation has been hotly debated. But his concept of emergence holds true: “The whole is not just the sum of its parts” because the emergent order will not arise if the parts simply coexist without interaction.

Complex interactions that Aristotle could not have anticipated, such as cell and solid organ transplantation and antimicrobial drug resistance, continue to stir up our biologic world. In this issue of Emerging Infectious Diseases, incidence of non-*Aspergillus* mold infections in hematopoietic cell and solid organ transplant recipients is increasing, and multiazole resistance in *Aspergillus fumigatus* associated with poor outcome in patients with invasive aspergillosis is now widespread in the Netherlands.

Long after Rembrandt painted Aristotle contemplating Homer, Nikos Kazantzakis wrote his version of The Odyssey. Having called modern humans to action against adversity and even against the inevitability of death, he engaged Homeric language to lead them in Aristotelian contemplation, not for any immediate resolution of perils in the world but as an end in itself: “I know not if I shall ever anchor.” Now the day’s work is done, “I collect my tools; sight, smell, touch, taste, hearing, intellect. Night has fallen …. I return like a mole to my home, the ground. Not because I am tired and cannot work. I am not tired. But the sun has set.”

## References

[1] Christian JL. Philosophy: an introduction to the art of wondering. Belmont (CA): Wadsworth Cengage Learning; 2009.

[2] Kazantzakis N. The Odyssey: a modern sequel. New York: Simon and Schuster; 1958.

[3] Park BJ, Pappas PG, Wannemuehler KA, Alexander BD, Anaissie EJ, Andes DR, Invasive non-*Aspergillus* mold infections in transplant recipients, United States, 2001–2006. Emerg Infect Dis. 2011;17:1855–64. 22000355 10.3201/eid1710.110087 PMC3311117

[4] Van der Linden JWM, Snelders E, Kampinga GA, Rijnders BJA, Mattsson E, Debets-Ossenkopp YJ, Clinical implications of azole resistance in *Aspergillus fumigatus*, the Netherlands, 2007–2009. Emerg Infect Dis. 2011;17:1846–54. 22000354 10.3201/eid1710.110226 PMC3311118

[5] Wallace W. “The legend and the man,” in the world of Rembrandt: 1606–1669. New York: Time-Life Library of Art; 1968.

